# Comment on “Incidental interictal epileptiform discharges in infants with nonepileptic events” by Montenegro et al.

**DOI:** 10.1111/epi.18686

**Published:** 2025-12-12

**Authors:** Margitta Seeck, Fábio A. Nascimento, Selim Benbadis, William O. Tatum, Elaine Wirrell, Georgia Ramantani, Sandor Beniczky

**Affiliations:** ^1^ Department of Clinical Neurosciences University Hospital of Geneva Geneva Switzerland; ^2^ Department of Neurology Washington University School of Medicine St. Louis Missouri USA; ^3^ Department of Neurology, Comprehensive Epilepsy Program University of South Florida and Tampa General Hospital Tampa Florida USA; ^4^ Mayo Clinic College of Medicine & Health Sciences Comprehensive Epilepsy Center Jacksonville Florida USA; ^5^ Division of Child and Adolescent Neurology and Epilepsy, Department of Neurology Mayo Clinic Rochester Minnesota USA; ^6^ Department of Neuropediatrics University Children's Hospital, and University of Zurich Zurich Switzerland; ^7^ Department of Clinical Neurophysiology Aarhus University Hospital Aarhus Denmark; ^8^ Aarhus and Danish Epilepsy Center Dianalund Denmark

To the Editors:

We read with interest the article by Montenegro et al.,^1^ in which the authors evaluated the frequency of incidental interictal epileptiform discharges (IEDs) on prolonged electroencephalograms (EEGs) of infants older than 28 days and up to 24 months of age who presented with nonepileptic paroxysmal events, diagnosed by video monitoring. Notably, all included patients had a normal neurologic examination and neurodevelopmental history, and none had a history of (provoked or unprovoked) seizures. The topic is important and clinically relevant, especially because it is understudied in this population compared to the literature in adults. We would like to offer our observations regarding the study.

The authors reported IEDs in 23.6% of 216 children without epilepsy (96% focal). We would like to raise concerns regarding the methodology employed to define IEDs. This reservation stems from two major points. First, the prevalence of incidental IEDs in this study was much higher than expected. Per meta‐analysis data, the prevalence of epileptiform abnormalities in children (age 1–17 years) is 2.45% (95% confidence interval = 1.41–4.21).^2^ Second, the examples of IEDs shown in figures 2 and 3 do not appear to be epileptiform to our eyes. The ones in figure 2 appear to be normal sharp/spiky fluctuations in the background activity (a.k.a. nonepileptiform sharp transients [NESTs]), which are the most common normal variants on EEGs.^3^ The example in figure 3 does not stand out as clearly epileptiform, although it is harder to appraise due to the high gain.

As outlined in several studies and established guidelines, six operational criteria are typically used to define IEDs (Figure 1),^4^, ^5^, ^6^ and at least four should be met for a sharp transient to be considered epileptiform. In the examples provided by in the article,^1^ fewer than four criteria are present, suggesting that these sharp transients are nonepileptiform (NESTs). In figure 2, example A meets criteria 1, 2, and 6; example B meets criteria 1 and 6; example C meets criteria 1, 2, and 6 (criterion 4 does not seem to be present; the slow waves intermixed with the sharp transients appear to be overlapping rhythms). Assuming these sharp transients were selected by the authors to represent the most illustrative incidental IEDs, we wonder whether the unshown examples may be even less convincingly epileptiform. Overcalling sharp transients as epileptiform may have led to an overestimation of the prevalence of IEDs in the studied population.

**FIGURE 1:**
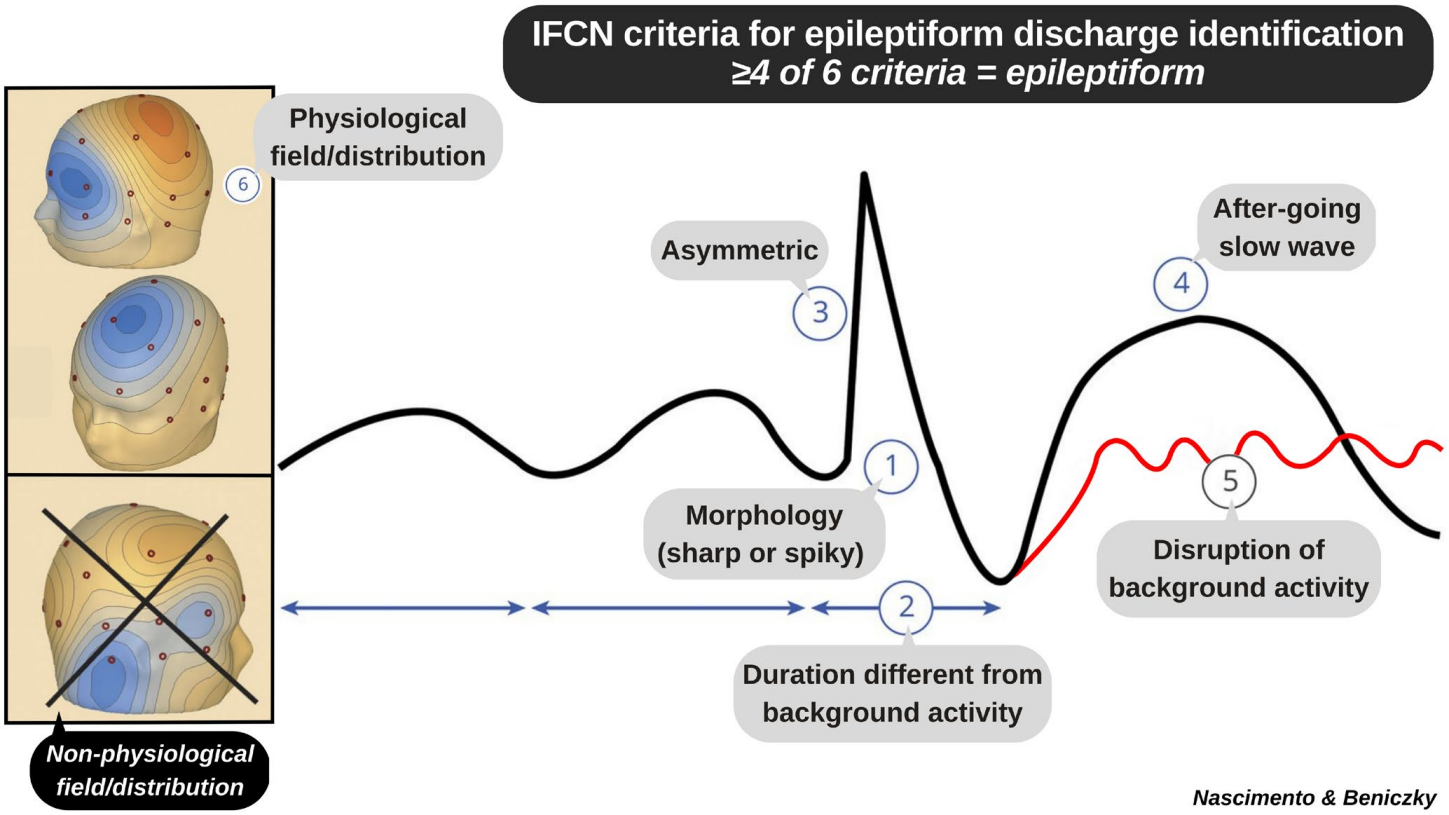
Adapted from Kural et al.,^4^ defining six criteria for interictal epileptiform discharges; at least four must be fulfilled for a pattern to qualify as epileptiform. IFCN, International Federation of Clinical Neurophysiology.

This study highlights the importance of accurate and reliable EEG interpretation, especially concerning the identification of IEDs given their major clinical implications. To achieve this goal, it is paramount that neurologists receive optimal training in EEG reading, ideally from EEG experts, in all contexts but particularly in countries where EEG training is not mandatory for those who read EEGs in clinical practice, such as the USA and many European countries,^7^, ^8^, ^9^ Furthermore, we stress the importance of applying rigorous operational criteria to avoid the practice of overreading, which is one of the major contributors to epilepsy misdiagnosis.^10^ The article does not reference the International Federation of Clinical Neurophysiology guidelines for the operational definition of IEDs, and the figures provided illustrate the consequences of this omission. Misclassifying benign sharp transients as IEDs risks overstating their clinical relevance and may lead to unnecessary concern or intervention.

## CONFLICT OF INTEREST STATEMENT

M.S. holds shares from Clouds of Care and dEEGtal, has received support from Angelini, Eisai, and Desitin, and is Editor‐in‐Chief of *Clinical Neurophysiology Practice*. E.W. is on data safety and monitoring boards for Encoded Therapeutics, GRIN Pharma, Neurocrine, and Acadia. Sa.B. is Editor‐in‐Chief of *Epileptic Disorders*. None of the other authors has any conflict of interest to disclose. We confirm that we have read the Journal's position on issues involved in ethical publication and affirm that this report is consistent with those guidelines.

## Data Availability

Data sharing not applicable to this article as no datasets were generated or analysed during the current study.

## References

[1] Montenegro MA , Tsuha M , Sattar S . Incidental interictal epileptiform discharges in infants with nonepileptic events. Epilepsia. 2025 May 20;66:3571–3577. 40394879 10.1111/epi.18461

[2] Aschner A , Kowal C , Arski O , Crispo JAG , Farhat N , Donner E . Prevalence of epileptiform electroencephalographic abnormalities in people without a history of seizures: a systematic review and meta‐analysis. Epilepsia. 2024 Mar;65(3):583–599. 38101821 10.1111/epi.17864

[3] Wüstenhagen S , Terney D , Gardella E , Meritam Larsen P , Rømer C , Aurlien H , et al. EEG normal variants: a prospective study using the SCORE system. Clin Neurophysiol Pract. 2022;7:183–200. 35865124 10.1016/j.cnp.2022.06.001 PMC9294211

[4] Kural MA , Duez L , Sejer Hansen V , Larsson PG , Rampp S , Schulz R , et al. Criteria for defining interictal epileptiform discharges in EEG: a clinical validation study. Neurology. 2020;94(20):e2139–e2147. 32321764 10.1212/WNL.0000000000009439 PMC7526669

[5] Kane N , Acharya J , Beniczky S , Caboclo L , Finnigan S , Kaplan PW , et al. Corrigendum to “A revised glossary of terms most commonly used by clinical electroencephalographers and updated proposal for the report format of the EEG findings. Revision 2017” [Clin. Neurophysiol. Practice 2 (2017) 170–185]. Clin Neurophysiol Pract. 2019;4:133. 31309168 10.1016/j.cnp.2019.06.001 PMC6606822

[6] Kane N , Acharya J , Benickzy S , Caboclo L , Finnigan S , Kaplan PW , et al. A revised glossary of terms most commonly used by clinical electroencephalographers and updated proposal for the report format of the EEG findings. Revision 2017. Clin Neurophysiol Pract. 2017;2:170–185. 30214992 10.1016/j.cnp.2017.07.002 PMC6123891

[7] Nascimento FA , Gavvala JR , Tankisi H , Beniczky S . Neurology resident EEG training in Europe. Clin Neurophysiol Pract. 2022 Aug 24;7:252–259. 36133398 10.1016/j.cnp.2022.08.001 PMC9483746

[8] Adornato BT , Drogan O , Thoresen P , Coleman M , Henderson VW , Henry KA , et al. The practice of neurology, 2000‐2010: report of the AAN member research subcommittee. Neurology. 2011 Nov 22;77(21):1921–1928. 22031533 10.1212/WNL.0b013e318238ee13

[9] Benbadis SR . “Just like EKGs!” should EEGs undergo a confirmatory interpretation by a clinical neurophysiologist? Neurology. 2013;80(1 Suppl 1):S47–S51. 23267045 10.1212/WNL.0b013e3182797539

[10] Oto MM . The misdiagnosis of epilepsy: appraising risks and managing uncertainty. Seizure. 2017;44:143–146. 28017581 10.1016/j.seizure.2016.11.029

